# Government audit, employee efficiency and labor cost stickiness

**DOI:** 10.1371/journal.pone.0291014

**Published:** 2023-09-01

**Authors:** Jia Li, Zhoutianyang Sun

**Affiliations:** 1 School of Accountancy, Tongling University, Tongling, Anhui, China; 2 School of Digital Economics and Management, Suzhou City University, Suzhou, Jiangsu, China; University of Baltistan, PAKISTAN

## Abstract

Does government audit affect employee allocation of enterprises? Using the quasi natural experiment and differences-in-differences(DID) model, this paper empirically tests the impact of government audit on employees efficiency and labor cost stickiness of state-owned enterprises (SOEs) in China. The results show that after the implementation of government audit, excess employees and labor cost stickiness of the audited enterprise is significantly reduced, which confirms the supervision and governance function of government audit on employee efficiency of SOEs. However, labor costs of the audited enterprise are not significantly reduced. These indicate from the side that the compensation system of SOEs conforms to market mechanisms, and there is no problem of employee over payment in China. This study enriches the relevant literature on the impact of government audit on employee policy of SOEs, and also provides empirical support for the full coverage of government audit.

## 1. Introduction

Employee allocation of state-owned enterprises is always a hot research topic in China. As the core content of enterprise element allocation, the ideal employee scale should be at its optimal quantity, contributing to the profit or value maximization goals, and can dynamically adjust according to the demand of production services and the size of allocated capital. However, due to political connection, state-owned enterprises (SOEs) bear more social burdens [1, 2]. SOEs tend to employ more employees, leading to serious redundancy and low employee efficiency [3, 4]. In order to achieve the political goal of full employment and stable economic development, SOEs not only hire more employees, but also retain redundant employees in the economic depression, so that their employee redundancy is greater than that of non-SOEs [5]. Government gives more tax incentives or financial subsidies to SOEs that bear the policy burden of excessive employees, and management may also ask government to give enterprises low interest loans, tariff protection and other additional policy preferences [6, 7]. However, while the policy tasks of SOEs bring various preferential policies, they also bring negative economic consequences to enterprises [1]. Excess employees not only directly increase the overall labor costs and management expenses, but also reduce the effectiveness of executive compensation contracts, and induce executives to seize excess compensation [4] .etc., which bring higher equity agency costs [8].

In addition to the size of employees, the employment policy of enterprises is mainly manifested in employee compensation. Although SOEs have remuneration controls in China, the << Provisions on the Composition of Total Wages>> doesn’t specify that total wages include transfer income, labor protection costs, etc, which allows SOEs pay benefits to their employees through these gray names. The welfare of SOEs are always higher than that of other enterprises [9, 10], which increases social costs of enterprises and reduces economic efficiency. Redundant employees and high wages and benefits together lead to high labor costs, reducing the competitiveness of SOEs. But high wages and benefits can also attract high-quality employees to join the enterprise, encourage employees to work hard, and increase investment in special human capital [11, 12]. In addition, the largest shareholder of SOEs is virtually empty, and there is a problem of owner vacancy. Management often form "insider control" to facilitate the transfer of benefits. Therefore, excessive wages and benefits may be the manifestation of serious agency problems. Management may not try to dismiss employees and reduce the wages and benefits when sales decline, which not only lead to high labor costs to reduce performance, but also form serious labor cost stickiness to magnify operational risk [3, 13, 14]. Labor cost stickiness is an important part of cost stickiness. Labor cost stickiness is related to the utilization efficiency of capitals, and significantly affects the performance and business risk of enterprises.

Although there are many literature on the impact of corporate internal governance on excess employees and labor costs, there is very few literature on the impact of external supervision represented by government audits on employee efficiency and labor costs of SOEs. Government audit of SOEs is the most complex and comprehensive audit type in all audits [15]. It is not only the audit of authenticity, legitimacy and effectiveness of financial revenues and expenditures, but also the audit of economy, efficiency, effectiveness, fairness and environment of business activities, in which the economic audit is the basis and the efficiency audit is the core [16]. Some literature These literature are published in Chinese domestic journals in Chinese. confirm the corporate governance effect of government audit, which can reduce the agency problems of management. For example, government audit can improve the operating efficiency, restrain earnings management, reduce excessive non-pecuniary compensation of management, improve the internal control quality, and so on. However, the impact of government audit on employee efficiency and labor costs is still unclear, which is a key operation and management activity of SOEs. As a strong government supervision measure, government audit has strong independence and strong information mining ability, and can reveal management problems in SOEs. With the economic efficiency audit becoming the focus of government audit, auditors naturally pay attention to the problem of employee efficiency, and reveal the problems of personnel, wages and benefits of enterprises. Does such external supervision effect the size of employees and labor costs of SOEs? that is, whether government audit can reduce excess employees, labor cost and labor cost stickiness of SOEs? The research on these problems and the development of relevant theories can not only fill the gap in the existing literature, but also have important significance for harnessing the supervision and governance functions of government audit, reducing agency problems within management and improving management efficiency of SOEs.

This paper finds that government audit reduces excess employees and labor cost stickiness by strengthening the incentive mechanism, and plays the role in improving corporate operation and promoting preservation and appreciation of the value of SOEs. Government audit strengthens the incentive mechanism, but doesn’t reduce labor costs. These indicate from the side that the compensation system of SOEs is relatively reasonable. Therefore, the possible marginal contribution of this paper is: (1) We analyze the impact of government audit on excess employees of SOEs, and find that external supervision from the government can effectively reduce excess employees and improve the resource utilization efficiency of enterprises. This paper enriches the literature on government regulation and enterprise operational efficiency. (2) We explore the impact of government audit on labor costs, and find that government audit doesn’t significantly reduce labor costs, but reduces labor costs stickiness, which may indicate that labor costs of SOEs are relatively reasonable. By comparing one study [9] that there is too many labor costs in SOEs, we can see that after years of reform and governance, SOEs have established market-oriented compensation structures, more in line with market mechanisms.(3)The intermediary effect of government audit strengthening incentive mechanism urges management to actively improve employee efficiency and effectively adjust employee compensation, indicating that government audit reduces the agency problems of management in state-owned enterprises. These discovery enlighten the new path of government audit affecting SOEs, and also show the importance of incentive mechanism. This paper provides an empirical basis for government audit to improve the incentive mechanism of SOEs.

Other contents are arranged as follows: The second part is the institutional background and hypothesis. After the institutional introduction and theoretical analysis, the research hypothesis are proposed. The third part is the research design, including sample selection and empirical design. The analysis of empirical results is the fourth part, the fifth part is the further analysis: mechanism test, and the sixth part is the conclusion and enlightenment.

## 2. Institutional background, theoretical analysis and hypothesis

### 2.1 Institutional background of government audit

As the "immune system" of national governance system, government audit supervision has the functions of prevention, disclosure and resistance, which is an independent and professional supervision, as well as a mandatory economic supervision activity. Article 91 of << Constitution of the People’s Republic of China>> endows the National Audit Office of State Council with the responsibility and power to audit SOEs. The audit of SOEs is the important contents of government audit. Every year, government audit needs to supervise SOEs, issue audit announcements, and make audit decisions according to law, or put forward punishment suggestions to relevant institutions. In recent years, the Central Committee of Communist Party of China and State Council have issued many important documents, in order to constantly strengthen the position and role of government audit in the economic supervision of SOEs and realize the preservation and appreciation of state-owned capital. The <<Guiding Opinions on Deepening the Reform of State owned Enterprises>> issued in 2015 puts forward the transformation of state-owned assets supervision to focus on "capital management", and implement full audit coverage of state-owned enterprise. The <<Several Opinions on Deepening the Audit and Supervision of State owned Enterprises and State owned Capital>> issued in 2017 emphasizes that government audit should increase the rectification and accountability of problems found in the audit, and fully plays the role of government audit in the national supervision system.

With the in-depth development of government audit and the continuous expansion of audit content, audits on state-owned enterprise by the National Audit Office have entered "comprehensive development stage focusing on economic responsibility audit". According to the relevant policy documents, the contents of economic responsibility audit include: "The quality of enterprise assets, profitability, debt risk, development capacity and other financial performance; enterprise development strategic planning, operation decision-making mechanism, internal risk control, human resources construction and other management performance; the completion of relevant target responsibility; the preservation and appreciation of state-owned capital and the return turned over; the investment, construction, management and benefits of important projects; the soundness and operation of corporate governance structure". The evaluation of government audit on the performance of economic responsibility is basically generated by comparing the evaluation criteria with evaluation indicator. It has the distinctive characteristics of performance appraisal, and takes audit results as an important basis for the rewards, punishments, appointment and dismissal of management, which can guide senior executives to link their own interests with the interests of enterprises, and actively pay attention to business performance. Therefore, government audit strengthens the incentive mechanism, and realizes the transformation of SOE executives from focusing on political future to business performance. In addition, government audit can also comment on management, finance and development strategies, and make suggestions to promote the improvement of operational performance of SOEs. Employee efficiency is an important factor that affects the operational performance of enterprises, thus becoming the focus of government audit and management attention.

### 2.2 Proposing hypothesis

The central and local governments at all levels implement preferential fiscal and tax policies for SOEs, and give institutional inducements or direct intervention in their employee employment policies to achieve the goal of full employment. Enterprises that have undertaken policy tasks such as excessive employees, need government to give them a certain degree of tax incentives and financial subsidies, and may also require government to give them low interest loans, tariff protection and other policy preferences [6, 7]. This connection between SOEs and the government distorts the allocation of enterprise resources, making SOEs have much excess employees, but at the same time, they also gain political rent-seeking benefits, such as more loan opportunities, financial subsidies and tax incentives, etc. Such explicit or implicit communication and cooperation are reduced because of the independence and deterrent effect of government audit. By standardizing the use of power, government audit continuously reduces the space of political rent-seeking, thus making management abandon the behavior of political rent-seeking. The power governance role of government audit breaks the tacit relationship between government and enterprises, restores the allocation of market resource, and then reduces excess employees of SOEs.

Redundant employees of SOEs not only bring high equity agency costs, which seriously reduce employee efficiency, but also increase labor costs and administrative expenses, which have a negative impact on corporate performance [1, 5]. As the economic efficiency becoming the focus of government audit, when the National Audit Office audits the economy, efficiency and effectiveness of enterprise activities, and auditors naturally pay attention to the efficiency of human resources. With more professional knowledge and stronger ability of information mining and analysis, auditors can identify the abnormal relationship between costs and benefits, judge the effectiveness of human resource allocation, and find departments with low employee efficiency. Moreover, auditors can comment on operation management, human resource construction, etc., put forward governance suggestions, and urge management to reduce redundant employees, which can improve the operating efficiency and performance of SOEs. In addition, government auditors announce audit results, expose the problems For example, Announcement No. 14 of 2014 "Audit Results of the Financial Receipts and Payments of China Weaponry Equipment Group Co., Ltd. in 2012" points out that the human resource management function of Chongqing Chang’an was idle for a long time. Announcement No. 41 of 2018 "Audit Results of the Assets, Liabilities, and Profits and Losses of China CITIC Group Co., Ltd. in 2016" points out that the proportion of information technology personnel doesn’t meet regulatory requirements. And so on. of audited SOEs in the above aspects, and strengthen the deterrent to senior executives of SOEs. The social concerns caused by audit announcements also exert pressure on senior executives, prompting them to take corrective measures to reduce excess employees.

Government audit pays attention to the quality of financial reports, which reduces earnings management and other behaviors that damage the quality of accounting information, and improves the transparency of enterprises [17]. Accounting information is closely related to the performance appraisal, and is also the basis of appointment, dismissal, rewards and punishments of management. High quality accounting information can help to form good incentive and restraint mechanism, and urge management to make the best efforts to create the performance of SOEs. Therefore, government audit plays the information governance role to ensure the authenticity and reliability of accounting information, which provides condition for the effective implementation of management incentive mechanism and strengthens the incentive function. On the other hand, some decisions of SOEs are based on social and political goals, which leads to the neglect of operating efficiency by management to a certain extent. Government audit can play good guiding role, correct some behaviors deviating from enterprise value, and urge management to pay more attention to business performance. In addition, government audit can improve internal system construction, and reduce power rent-seeking and corruption [16]. These can also encourage management to work actively, and improve the corporate performance by reducing excess employees, in order to obtain rewards and market reputation. To sum up, the hypothesis H1 of this paper is proposed:

**H1: Government audit significantly reduces excess employees of state-owned enterprises (SOEs)**.

SOEs shoulder more political tasks and social responsibilities [1, 2], and have broader social goals, which makes the enterprise bear more labor costs, such as maintaining employment, excess welfare, etc. Government audit reduces political connection to a certain extent and strengthens the incentive mechanism of SOEs. The motivation for management to improve performance by cutting costs also enhances. As the main cost expenditure, labor costs directly affect the net profit of enterprises. Management can improve the net profit by reducing labor costs and related expenses. When the incentive mechanism is strengthened after government audit, in order to pursue profits and create value, management may actively reduce labor costs, resulting in the overall reduction of labor costs of audited SOEs. Therefore, after the intervention of government audit, labor costs of SOEs are significantly reduced.

According to the efficiency wage theory, high salary has a positive impact on the performance of enterprises, and high salary can motivate employees to work hard [12, 18]. Moreover, the specificity of human capital also makes salary incentive more important for the improvement of employees’ work efficiency. On the one hand, the formation and development of human capital are inseparable from specific work experience, which requires employees to pay more costs, and may also pose a risk of being locked up. Employees are often under-invested in human capital, while high salary can encourage employees to strengthen investment in human capital related to the enterprise, and also attract outstanding external employees to join the enterprise [11, 18]. On the other hand, the ownership of human capital belongs to individuals, and enterprises only have the right to use. Therefore, working attitude of employees effects the use efficiency of human capital, and high salary can encourage employees to work hard. In addition, there is not only working relationship of explicit contract, but also private relationship of implicit contract between management and employees. Improving and maintaining employee compensation can not only establish good psychological contract with employees, but also reduce the psychological pressure brought by competition. Therefore, when the incentive mechanism is strengthened after government audit, management still maintain employees’ wages and benefits, and labor costs don’t decrease significantly. Therefore, after the intervention of government audit, labor costs of SOEs isn’t significantly affected. To sum up, we propose the hypothesis H2:

**H2a: Government audit significantly reduces labor costs of state-owned enterprises (SOEs)**.
**H2b: Government audit doesn’t significantly reduce labor costs of state-owned enterprises (SOEs)**.

The ownership structure and executive appointment of SOEs are quite different from those of non-SOEs represented by private enterprises and foreign-owned enterprises, which are characterized by high political relevance. SOEs should not only pursue economic goals, but also have more political tasks and social responsibilities [1, 2], such as stabilizing economy and promoting employment, etc. Therefore, when sales drops, political connection is not conducive to the reduction of salaries or layoffs, showing higher labor cost stickiness [3]. In addition, there are more serious principal-agent problems in SOEs, and "insider control" is serious. In order to pursue high social status and resources that can be controlled by individuals, management may expand the investment scale as much as possible, and obtain the increase of outputs and profits through extensive input of production factors. Even if sales of the enterprise decline, management may not easily reduce resources and benefits they enjoy, not actively make efforts to carry out effective cost management, which further strengthens the problem of cost stickiness of SOEs [3]. The following focuses on the difference between labor cost stickiness of SOEs after government audit and those not audited.

Government audit can correct some activities that deviating from enterprise value, alleviate the agency problem of management, and encourage managers to work hard in creating performance for SOEs. Government audit includes not only the authenticity and legitimacy of financial revenue and expenditure, but also the economy and efficiency of the performance, which has a distinctive feature of performance appraisal. The attention of government audit to financial contents prompts management to reduce earnings management to whitewash financial statements, and ensures the reliability of accounting information. Moreover, the economic content audit achieves the performance evaluation by comparing the evaluation criteria with the evaluation indicator, and then strengthens the incentive mechanism. Under the effect of the incentive mechanism, management may improve the performance by reducing labor costs when sales situation is not good, because cutting labor costs can improve the utilization efficiency of capital, and then improve the enterprise profits. Therefore, after the intervention of government audit, labor cost stickiness of SOEs is significantly reduced.

A large number of literature on employee motivation focus on the field of labor economics, and most believe that compensation level related to performance has a positive impact on business performance. For example, one study [11] believes that performance-based compensation can motivate employees to work hard. Government audit reduces the adverse impact of political connection, so that SOEs can reduce labor costs by reducing employee compensation in a reasonable way when sales situation is poor. At the same time, the intervention of government audit strengthens the incentive mechanism for management, and since the linkage between employee compensation and performance has a positive impact on future profits [11, 18], management may strengthen the correlation between employee compensation and performance. Audited SOEs strictly adjust the compensation of employees according to sales of enterprises. When sales decrease, management are more likely to significantly reduce employee compensation, causing a decrease in employee compensation stickiness. Therefore, after the intervention of government audit, employee compensation stickiness of SOEs is significantly reduced. To sum up, we propose the hypothesis H3:

**H3: Government audit significantly reduces labor cost stickiness and employee compensate stickiness of state-owned enterprises (SOEs)**.

## 3. Research design

### 3.1 Sample selection and data sources

Since Chinese new accounting standards have been implemented since 2007, in order to maintain the consistency of data statistics, this paper uses the data of A-share companies from 2007 to 2018 as empirical sample. Firstly, selecting the sample of audited enterprises. The sample selected in this paper is the audited enterprises whose government audit announcement is from 2010 to 2018. According to the statistics released by National Audit Office, 157 state-owned enterprise groups have been audited in the sample year. Among them, 27 enterprise groups are repeatedly audited twice, and 3 enterprise groups are repeatedly audited three times. Combined with the information in the CSMAR(China Stock Market & Accounting Research Database) and iFinD database, we find A-share listed enterprises whose direct controller and actual controller are audited SOEs. Excluding financial enterprises and data missing observations, 1172 observation enterprises are left. Secondly, selecting the sample of not-audited enterprises. Since National Audit Office only audits SOEs, and there are significant differences between SOEs and non-SOEs in China. Therefore, it is not appropriate to take private enterprises as the control group, and we take SOEs as the control group. In order to test hypothesis H1, H2 and H3, it is necessary to determine not-audited enterprises in corresponding years. In the first step, according to enterprise attributes in the database, we select SOEs which are not audited from 2007–2018. In the second step, financial enterprises and data missing observation are eliminated. Finally, 9777 observation of not audited enterprises are obtained. The total number of samples is 10949.

### 3.2 Variable definition and model design

#### 3.2.1 Government audit and excess employees

According to the existing research, the most important factors that determine the size of employees are firm size, capital density, firm growth and industry characteristics. In addition, firm profitability and financial situation also significantly affect firm’s employee demand. Therefore, the normal number of employees can be estimated based on these six factors, and the difference between the actual number and the normal number of employees represents the degree of excess employees. We use the linear method to estimate excess employees by the following formula:

$$emp_{it}=\beta_{0}+\beta_{1}Lnsize_{it}+\beta_{2}Ppe_{it}+\beta_{3}Grow_{it}+\beta_{4}Roa_{it}+\beta_{5}Lev_{it}+\varepsilon_{it}$$ (1)

Where, *emp*_*it*_ represents the actual number of employees, which is expressed by dividing the number of employees by total assets at the end of the year and multiplied by 100000. *Lnsize* is firm size, measured by the natural logarithm of total assets at the end of the period. *Ppe* is the relative size of fixed assets, representing capital density, which is measured by the ratio of fixed assets to total assets at the end of the period. *Grow* is the growth rate of operating revenue, representing the growth of the enterprise, which is measured by subtracting previous operating revenue from current operating revenue and dividing it by previous operating revenue. *Roa* is the net profit rate of assets, representing the profitability, which is measured by dividing the current net profit by total assets at the end of the period. *Lev* is financial leverage, representing financial situation, which is measured by dividing total liabilities by total assets at the end of the period. Carry out linear regression of the above Eq (1) by industry, and use the residual *ε*_*it*_ to represent excess employees. Although residuals after model regression has no clear economic meaning, the relative size of residuals can measure the degree of redundant employees. Compared with the company with small residuals, the company with large residuals have more redundant employees. Compared with the company with negative residuals, the company with positive residuals have more redundant employees. In a word, the larger residual value *ε*_*it*_ represents the higher degree of redundant employees. Since the verification of hypothesis H1 needs to compare the change of excess employees in different years, and the mean value of residuals regressed by year is equal in each year (the expected mean value is 0), so annual regression is not used here. In order to make empirical results more reliable, we obtain two indicators of excess employees. According to the above formula, residuals are *Exemp*1 calculated regardless of industry, and *Exemp*2 calculated by industry.

After calculating the index of excess employees, we refer to the existing research and use the double difference model for empirical analysis. Model (2) is used to test the change of excess employees of SOEs after the government audit intervention, as shown below. *List*_*i*_ is used as government audit dummy variable. In the sample period, if the group to which listed company belongs has been audited, it is taken as 1, otherwise it is taken as 0. *Postlist*_*it*−1_ indicates the year before or after government audit intervention. For the year of government audit intervention and subsequent years, *Postlist*_*it*_ is 1, and others are 0. Explained variables are the measurement variables of excess employees, *Exemp*1 and *Exemp* 2. Explanatory variable *Postlist* indicates the year after the government audit intervention. The control variables include total asset yield *Roa*, capital density *Cap*, tobin Q value *Q*, enterprise size *Lnsize*, shareholding ratio of top three institutions *Threeins*. *Age* refers to the time when the company is listed, which is the number of days from the listing date to the end of the period divided by 360. Inventory changing *Invchange*, the ratio of sum of cash and cash equivalents to total assets *Cash*, cash dividend *Dividend*, and *Grow* representing the growth rate of operating incomes.


$$\begin{array}{l} Exemp_{it+1}=\beta_{0}+\beta_{1}List_{i}+\beta_{2}Postlist_{it}+\beta_{3}Lnsize_{it}+\beta_{4}Roa_{it}+\beta_{5}Threeins_{it}\\ +\beta_{6}Age_{it}+\beta_{7}Cap_{it}+\beta_{8}Q_{it}+\beta_{9}Invchange_{it}+\beta_{10}Cash_{it}+\beta_{11}Dividend_{it}\\ +\beta_{12}Grow_{it}+\sum Year+\sum Industry+\varepsilon_{it} \end{array}$$ (2)

#### 3.2.2 Government audit and labor costs

In order to test the impact of government audit on labor costs, we adopt four methods to measure labor costs. The main form of labor costs in enterprises is employee compensation. We refer to the calculation method of employee compensation by [19], use the "cash paid to and for employees" disclosed in the cash flow statement to subtract total executive compensation, divided by the total number of employees excluding senior executives, and then take the natural logarithm, recorded as *Labcost1*. In addition, we also use three other indicators to test hypothesis H2, namely, the natural logarithm of "cash paid to and for employees", recorded as *Labcost2*; "Cash paid to and for employees" divided by total operating costs, recorded as *Labcost3*; "Cash paid to and for employees" divided by average total assets, recorded as *Labcost4*. We use double difference model for reference and adopt the following model (3) to test. Explained variables are the measurement variables representing labor costs, which are *Labcost1*, *Labcost2*, *Labcost3* and *Labcost4* in turn, and explanatory variable is *Postlist*, which indicates the year before or after government audit intervention. The control variables include total asset yield *Roa*, capital density *Cap*, enterprise scale *Lnsize*, shareholding ratio of top three institutions *Threeins*, inventory changing *Invchange*, asset liability ratio *Lev*, operating income growth rate *Grow*, fixed asset scale *Ppe*.


$$\begin{array}{l} Labcost_{it}{}_{-1}=\beta_{0}+\beta_{1}List_{i}+\beta_{2}Postlist_{it}+\beta_{3}Roa_{it}+\\ \beta_{4}Cap_{it}+\beta_{5}Lnsize_{it}+\beta_{6}Threeins_{it}+\\ \beta_{7}Invchange_{it}+\beta_{8}Lev_{it}+\beta_{9}Grow_{it}+\beta_{10}Ppe_{it}+\\ \sum Year+\sum Industry+\varepsilon_{it} \end{array}$$ (3)

#### 3.2.3 Government audit and labor cost stickiness

Labor cost stickiness refers to the stickiness of total employee compensate. The latter part proves the stickiness of per capita compensate. Our empirical model draws on the sticky cost model of [20] and [21], and is modified by the double difference method. Firstly, the model links the change logarithm (*Lnlabcost*_*it*_)of labor costs with the change logarithm(*Lnsale*_*it*_)of sales in the same period, as shown below.


$$Lnlabcost_{it}=\alpha_{0}+\alpha_{1it}Lnsale_{it}+\alpha_{2it}Lnsale_{it}Dec_{it}+\mu_{it}$$ (4.1)

Subscripts *i* and *t* represent the company and year respectively, and *Lnlabcost*_*it*_ represents the change rate of labor costs, i.e, *Lnlabcost*_*i*.*t*_ = *Ln*(*Labcost*_*i*.*t*_/*Labcost*_*i*.*t−1*_) as the explained Variable. Where, *Labcost* is the amount of "cash paid to and for employees" disclosed in the cash flow statement minus total executive compensation. *Lnsale*_*it*_ is the change logarithmic of sales, representing the change rate of main business incomes, and *Dec*_*it*_ is the dummy variable of main business income decline. If annual sales decrease, it is 1, otherwise it is 0. While *μ*_*it*_ is the error term, the average value is zero and it is independent of the explanatory variable. Coefficient *α*_1*it*_ represents the percentage increase in labor costs when sales increase by 1%. *α*_1*it*_ + *α*_2*it*_ represents the percentage decrease in labor costs when sales decrease by 1%, and labor cost stickiness coefficient *α*_2*it*_ reflects the degree of labor cost asymmetry between sales decline and growth. If *α*_1*it*_ is positive and *α*_2*it*_ is negative, labor costs are sticky. We introduce a hierarchical linear model in which the first-order result of the basic model Eq (4.1) is constructed as a function of the second-order model of explain variables and control variables.

We extend the basic cost stickiness model of [20] to allow government audit variables and other control variables to influence labor cost stickiness coefficient *α*_2*it*_. One study [22] finds that capital intensity and labor intensity have significant effects on cost stickiness. In capital-intensive or labor-intensity enterprises, cost stickiness is more serious. Therefore, we control capital intensity *Cap*_*it*_ of the enterprise, using the ratio between total assets and business incomes to express, and also control labor intensity *Emp*_*it*_, using total remuneration of employees divided by business incomes to express. In addition, *Dec*_*it*−1_ is a dummy variable. If sales decrease from t-2 to t-1, take 1. Otherwise, 0. *Dec*_*it*−1_ can represent optimistic or pessimistic expectation of management about future demand. Specifically, we introduce a two-level model to set the coefficients *α*_1*it*_ and *α*_2*it*_ in Eq (4.1) as functions of government audit *List*_*i*_ and *Postlist*_*it*−1_, capital intensity *Cap*_*it*_, labor intensity *Emp*_*it*_, dummy variable *Dec*_*it*−1_. By constructing, the average values of *v*_1_ and *v*_2_ are zero, independent of explanatory variables, as follows:

$$\alpha_{1it}=\beta_{1}+\beta_{2}Cap_{it}+\beta_{3}Emp_{it}+\beta_{4}Dec_{it-1}+\nu_{1}$$ (4.2a)

The *List*_*i*_ and *Postlist*_*it−1*_ variables are not included in the Eq (4.2a), because, on the one hand, government audit doesn’t affect the cost change when sales increase, and on the other hand, the problem of multicollinearity is avoided.


$$\alpha_{2it}=\beta_{5}+\beta_{6}List_{i}+\beta_{7}Postlist_{it-1}+\beta_{8}Cap_{it}+\beta_{9}Emp_{it}+\beta_{10}Dec_{it-1}+\nu_{2}$$ (4.2b)

In formula (4.2b), we use the double difference model. Our main interest is the coefficient *β*_7_ of *Postlist*_*it*_ * *Lnsale*_*it*_ * *Dec*_*it*_, which captures the impact of government audit on labor cost stickiness. If *β*_7_ is significantly positive, it means that labor cost stickiness of enterprises decreases after government audit. If *β*_7_ is significantly negative, it indicates that labor cost stickiness of enterprises increases significantly after government audit.

Combining formula (4.1) with formula (4.2), the following test formula can be obtained:

Lnlabcostit=α0+(β1+β2Capit+β3Empit+β4Decit−1)Lnsaleit+(β5+β6Listi+β7Postlistit−1+β8Capit+β9Empit+β10Decit−1)LnsaleitDecit+∑Year+t∑Industryj+εit (4.3)

Among them, subscripts *i*, *j*, and *t* represent company, industry and year respectively. The other variables have the same meaning as above. Finally, the *Industry* and *Year* dummy variables are added to control the impact of fixed effects of the year and industry respectively. *ε*_*it*_ represents the error term. Combining the above four formulas, we can get the following formula:

$$\varepsilon_{it}=\mu_{it}+\nu_{1}*Lnsale_{it}+\nu_{2}*Lnsale_{it}*Dec_{it}$$ (4.4)

The original error terms *μ*_*it*_, *v*_1_ and *v*_2_ from Eqs (4.1), (4.2a), and (4.2b) all have zero mean value and are independent of explanatory variables, so the synthetic error term *ε*_*it*_ is also zero mean. Therefore, OLS will produce unbiased and consistent estimates.

## 4. Empirical analysis

### 4.1 Descriptive statistical analysis

It can be seen from Table 1 that the average value of excess employees *Exemp*1 regardless of industry is -0.002, and the average value of excess employees *Exemp*2 by industry is -0.014, which is consistent with the existing research. The average value of *List* is 0.257, and the average value of *Postlist* is 0.125, which provides us with relatively reasonable data grouping, that is, the experimental group and the control group, which are convenient for comparative research and also provide good prerequisite for double difference. The average value of *Cash* is 0.155, indicating that the cash balance of SOEs during the sample period is relatively high. The average value of *Threeins* is 57.8%, indicating that the equity of SOEs is relatively concentrated. The average value of *Lev* is 0.531, higher than 0.5, indicating that SOEs have the task of deleveraging. The average value of *Dec* is 0.286, indicating that 28.6% of the observations of SOEs decline during the sample period. Many enterprises have experienced sales decline, which makes the sample have a reasonable quantitative basis to verify labor cost stickiness. In Table 2, it is found that not-audited SOEs are significantly higher than audited SOEs by comparing the estimated excess employees variable *Exemp*2.

**Table 1:** Description statistics of main variables.

variable	mean	sd	p25	p50	p75
*Exemp*1	-0.002	0.080	-0.047	-0.015	0.023
*Exemp*2	-0.014	0.204	-0.058	-0.030	0.009
*List*	0.257	0.437	0.000	0.000	1.000
*Postlist*	0.125	0.331	0.000	0.000	0.000
*Lnsize*	22.46	1.508	21.45	22.30	23.39
*Roa*	0.041	0.226	0.013	0.034	0.066
*Lev*	0.531	1.063	0.355	0.518	0.664
*Q*	1.993	25.01	0.642	1.161	2.027
*Invchange*	0.021	0.080	-0.005	0.006	0.033
*Cash*	0.155	0.122	0.072	0.122	0.201
*Dividend*	0.159	0.326	0.050	0.100	0.200
*Ppe*	0.280	0.207	0.111	0.237	0.417
*Threeins*	0.578	0.159	0.465	0.579	0.691
*Age*	2.463	0.626	2.233	2.646	2.894
*Cap*	3.071	20.76	1.162	1.758	2.887
*Emp*_*it*_	0.184	6.332	0.059	0.098	0.153
*Grow*	0.181	0.594	-0.025	0.098	0.247
*Labcost*1	11.50	0.664	11.11	11.49	11.86
*Labcost*2	19.42	1.487	18.42	19.32	20.32
*Labcost*3	0.128	0.093	0.062	0.105	0.166
*Labcost*4	0.070	0.069	0.034	0.058	0.092
*Lnlabcost*1	0.143	0.323	0.030	0.112	0.209
*Lncompen*	0.077	0.352	0.007	0.086	0.168
*Dec*_*it*_	0.286	0.452	0.000	0.000	1.000
*Gdpg*_*it*_	0.081	0.014	0.068	0.078	0.095

**Table 2:** Comparative analysis of excess employee(*Exemp*2) in SOEs that is audited and not audited.

year	Variables	postlist = 0		postlist = 1		MeanDiff
		N	Mean1	N	Mean2	
2009	*Exemp*2	308	-0.007	11	-0.048	0.040*
2010	*Exemp*2	250	-0.005	74	-0.034	0.029***
2011	*Exemp*2	220	-0.006	123	-0.017	0.012
2012	*Exemp*2	206	-0.010	144	-0.026	0.017**
2013	*Exemp*2	185	-0.010	165	-0.031	0.020***
2014	*Exemp*2	165	-0.010	189	-0.035	0.025***
2015	*Exemp*2	143	-0.015	212	-0.031	0.016**
2016	*Exemp*2	113	-0.013	243	-0.034	0.021***
2017	*Exemp*2	83	-0.012	273	-0.034	0.022***
total	*Exemp*2	2288	-0.006	1434	-0.031	0.025***

### 4.2 Model regression analysis

#### 4.2.1 Government audits and excess employees

The regression results of model (2) are shown in Table 3. It can be seen from the table that when *Exemp*1 is used as the explained variable, the regression coefficients of *Postlist* are significantly negative. The coefficient of *Postlist* in the regression without control variable is -0.014 at the 1% significance level, and the coefficient of *Postlist* in the regression with control variables is -0.017 at the 1% significance level. When *Exemp*2 is used as the explained variable, the coefficient of *Postlist* is also significantly negative. In the regression without control variables, the coefficient of *Postlist* is -0.026 at the 1% significance level, and in the regression with control variables, the coefficient of *Postlist* is -0.017 at the 1% significance level. It can be seen that after the intervention of government audit, excess employee of audited SOEs decrease significantly, and government audit improves employee efficiency, supporting the hypothesis H1. We also use the fixed effect model for regression, and results obtained are basically unchanged.

**Table 3:** Regression coefficient of government audit and excess employees.

VARIABLES	(1)	(2)	(3)	(4)
	*Exemp*1_*it*+1_	*Exemp*1_*it*+1_	*Exemp*2_*it*+1_	*Exemp*2_*it*+1_
*List*_*it*_	0.011***	0.008***	0.007**	0.007***
	(5.484)	(3.097)	(2.179)	(2.671)
*Postlist*_*it*_	-0.014***	-0.017***	-0.026***	-0.017***
	(-6.104)	(-6.789)	(-10.090)	(-6.242)
*Lnsize*_*it*_		0.011***		-0.003***
		(15.014)		(-4.012)
*Roa*_*it*_		0.112***		0.024
		(4.286)		(0.900)
*Threeins*_*it*_		-0.006		-0.018***
		(-1.085)		(-3.142)
*Cap*_*it*_		-0.006***		-0.006***
		(-5.727)		(-5.579)
*Q*_*it*_		-0.000		0.001
		(-0.103)		(1.033)
*Inventorycha*_*it*_		0.007		0.042***
		(0.888)		(4.966)
*Cash*_*it*_		0.029***		0.076***
		(3.860)		(9.793)
*Dividend*_*it*_		-0.002		-0.003
		(-0.779)		(-1.373)
*Grow*_*it*_		-0.004**		-0.004**
		(-2.466)		(-2.508)
*Year*	No-Control	Control	No-Control	Control
*Industry*	No-Control	Control	No-Control	Control
Constant	-0.003***	-0.243***	-0.013***	0.062***
	(-3.137)	(-14.200)	(-4.830)	(3.455)
Observations	10,949	6,027	10,949	6,027
R-squared	0.003	0.113	0.001	0.107

Robust t-statistics in parentheses

*** p<0.01,

** p<0.05,

* p<0.1.

#### 4.2.2 Government audit and labor costs

The regression results of model (3) are shown in Table 4. It can be seen that when *Labcost1*, *Labcost2*, *Labcost3* and *Labcost4* are used as the explained variables in turn, the coefficients of *Postlist* are not significant. It can be seen that after the intervention of government audit, labor costs of audited SOEs doesn’t change significantly, supporting the hypothesis H2b. This shows that although government audit intervention strengthens incentive mechanism for enterprise executives, executives don’t blindly reduce wages and welfare of employees for the sake of performance, so as to maintain the enthusiasm of employees. This also shows that wages and benefits of employees of SOEs are in line with market mechanisms, which can motivate employees. We also use fixed effect model for regression, and results are basically unchanged.

**Table 4:** Regression coefficient of government audit and labor costs.

VARIABLES	(1)	(2)	(3)	(4)
	*Labcost1*_*it*+1_	*Labcost2*_*it*+1_	*Labcost3*_*it*+1_	*Labcost4*_*it*+1_
*List*_*it*_	0.161***	0.054**	-0.001	0.002
	(4.795)	(2.140)	(-0.835)	(0.744)
*Postlist*_*it*_	0.035	0.001	-0.002	0.003
	(0.832)	(0.027)	(-1.136)	(1.054)
*Roa*_*it*_	0.426	0.931***	0.246***	0.125***
	(1.588)	(3.831)	(9.606)	(5.307)
*Cap*_*it*_	0.021*	-0.243***	0.001***	-0.012***
	(1.805)	(-20.758)	(3.422)	(-11.630)
*Lnsize*_*it*_	0.064***	0.931***	-0.001	-0.005***
	(4.412)	(63.261)	(-0.755)	(-4.552)
*Threeins*_*it*_	0.471***	0.271***	0.011*	0.024***
	(4.094)	(3.440)	(1.911)	(3.931)
*Inventorycha*_*it*_	-0.197	-0.140	0.010***	0.017**
	(-1.450)	(-1.326)	(2.762)	(2.477)
*Lev*_*it*_	-0.121	0.012	-0.023***	0.020***
	(-1.218)	(0.156)	(-3.636)	(3.055)
*Grow*_*it*_	-0.015	-0.049***	-0.001**	0.007***
	(-0.810)	(-2.901)	(-2.105)	(3.867)
*Emp*_*it*_	-0.108	5.940***	1.012***	0.414***
	(-0.522)	(24.270)	(37.214)	(15.634)
*Age*_*it*_	0.076***	0.061***	-0.003***	0.006***
	(3.709)	(3.630)	(-2.982)	(3.819)
*Q*_*it*_	0.008	-0.021	-0.003	-0.002
	(0.899)	(-1.057)	(-1.123)	(-0.942)
*Ppe*_*it*_	-0.307***	0.093	0.005*	0.002
	(-3.434)	(1.539)	(1.666)	(0.455)
*Cash*_*it*_	0.365***	0.128	-0.000	0.015*
	(2.608)	(1.249)	(-0.075)	(1.903)
*Year*	Control	Control	Control	Control
*Industry*	Control	Control	Control	Control
Constant	9.365***	-1.894***	0.043	0.140***
	(29.426)	(-5.069)	(0.964)	(4.701)
Observations	9,082	9,089	9,089	9,089
R-squared	0.284	0.937	0.942	0.651

Robust t-statistics in parentheses

*** p<0.01,

** p<0.05,

* p<0.1

#### 4.2.3 Government audit and labor cost stickiness

The regression results of model (4.3) are shown in Table 5. Due to the problem of multicollinearity, we try to remove the relevant variables for regression analysis and conduct VIF test. The relevant VIF test is shown in Table 6. There is a serious multicollinearity problem in model 1. When *Emp*_*it*_ * *Lnsale*_*it*_ is removed for model 2, the problem of multicollinearity still exists. In Model 3, *Cap*_*it*_ * *Lnsale*_*it*_ is also removed, and there is no problem of multicollinearity. In Model 4, *Emp*_*it*_ * *Lnsale*_*it*_ and *Cap*_*it*_ * *Lnsale*_*it*_ * *Dec*_*it*_ are removed, and there is no multicollinearity problem. The results of the above four models are shown in Table 6. It can be seen that the coefficient of *Postlist*_*it*−1_ * *Lnsale*_*it*_ * *Dec*_*it*_ is significantly positive in the four models, which are 0.214, 0.201, 0.193 and 0.200 respectively. In the years after government audit intervention, labor cost stickiness of listed companies is significantly reduced, supporting the hypothesis H3. Government audit promotes the dynamic adjustment of labor costs, with market-oriented operation, and improves management efficiency of SOEs.

**Table 5:** Regression coefficient of labor labor stickiness equation.

VARIABLES	(1)	(2)	(3)	(4)
	*Lnlabcost*_*it*_	*Lnlabcost*_*it*_	*Lnlabcost*_*it*_	*Lnlabcost*_*it*_
*Lnsale*_*it*_	0.955***	0.674***	0.546***	0.650***
	(22.206)	(70.428)	(52.335)	(70.454)
*Cap*_*it*_ * *Lnsale*_*it*_	-0.023***	-0.009***		-0.001***
	(-21.109)	(-9.397)		(-10.091)
*Emp*_*it*_ * *Lnsale*_*it*_	1.485***		0.995***	
	(27.168)		(20.076)	
*Dec*_*it*−1_ * *Lnsale*_*it*_	-0.202***	-0.195***	-0.225***	-0.203***
	(-17.155)	(-15.926)	(-18.637)	(-16.607)
*Lnsale*_*it*_ * *Dec*_*it*_	-1.281***	-0.600***	-0.472***	-0.577***
	(-12.417)	(-25.926)	(-20.290)	(-25.004)
*List*_*i*_ * *Lnsale*_*it*_ * *Dec*_*it*_	-0.077	-0.075	-0.066	-0.074
	(-1.421)	(-1.336)	(-1.197)	(-1.306)
*Postlist*_*it*−1_ * *Lnsale*_*it*_ * *Dec*_*it*_	0.214***	0.201***	0.193***	0.200***
	(3.301)	(3.017)	(2.941)	(2.983)
*Cap*_*it*_ * *Lnsale*_*it*_ * *Dec*_*it*_	0.022***	0.009***	-0.001***	
	(20.456)	(8.877)	(-9.836)	
*Emp*_*it*_ * *Lnsale*_*it*_ * *Dec*_*it*_	-1.483***	0.001***	-0.994***	0.001***
	(-27.144)	(7.556)	(-20.055)	(7.973)
*Dec*_*it*−1_ * *Lnsale*_*it*_ * *Dec*_*it*_	0.438***	0.432***	0.463***	0.446***
	(15.762)	(14.942)	(16.249)	(15.387)
*Year*	control	control	control	control
*Industry*	control	control	control	control
Constant	0.019	0.011	0.007	0.013
	(1.495)	(0.847)	(0.556)	(1.025)
Observations	10,195	10,195	10,195	10,195
R-squared	0.485	0.439	0.456	0.435

t-statistics in parentheses

*** p<0.01,

** p<0.05,

* p<0.1

**Table 6:** The VIF test of labor labor stickiness regression equation.

Variable	(1)	(2)	(3)	(4)
	VIF	VIF	VIF	VIF
*Lnsale*_*it*_	106.58	4.31	4.59	2.97
*Cap*_*it*_ * *Lnsale*_*it*_	89.05	83.9		4.2
*Emp*_*it*_ * *Lnsale*_*it*_	4.04		3.45	
*Lnsale*_*it*_ * *Dec*_*it*_	2.56	2.41	2.49	2.39
*Lnsale*_*it*_ * *Dec*_*it*_	201.93	7.99	8.05	7.76
*List*_*i*_ * *Lnsale*_*it*_ * *Dec*_*it*_	6.33	6.32	6.32	6.31
*Postlist*_*it*−1_ * *Lnsale*_*it*_ * *Dec*_*it*_	4.59	4.34	4.34	4.34
*Cap*_*it*_ * *Lnsale*_*it*_ * *Dec*_*it*_	90.55	85.27	4.26	
*Emp*_*it*_ * *Lnsale*_*it*_ * *Dec*_*it*_	8.01	6.27	7.51	6.12
*Dec*_*it*−1_ * *Lnsale*_*it*_ * *Dec*_*it*_	2.76	2.57	2.57	2.57
*Year*	.....	.....		
*Industry*	....	.....		
Mean VIF	25.24	8.35	2.84	2.69

From hypothesis H2b and hypothesis H3, it can be seen that after government audit, labor costs of audited SOEs don’t change significantly, but labor cost stickiness decreases significantly. It can be seen that after government audit strengthening incentive mechanism, management make great efforts to improve the performance and reduce labor cost stickiness, but not reduce labor costs, which also shows from the side that wages and benefits of employees of SOEs is reasonable on the whole.

In order to prevent the impact of multicollinearity on empirical results, we try to remove the relevant variables for regression analysis and conduct VIF test. The relevant VIF test is not listed. There is a serious multicollinearity problem in model 1. When *Emp*_*it*_ * *Lnsale*_*it*_ is removed for model 2, there is still a problem of multicollinearity. In model 3, *Cap*_*it*_ * *Lnsale*_*it*_ is also removed, and there is no problem of multicollinearity. In model 4, *Emp*_*it*_ * *Lnsale*_*it*_ and *Cap*_*it*_ * *Lnsale*_*it*_ * *Dec*_*it*_ are removed, and there is no multicollinearity problem. The results of the above four models are shown in Table 7. It can be seen that the coefficients of *Postlist*_*it*−1_ * *Lnsale*_*it*_ * *Dec*_*it*_ are all significantly positive in the four models. After the government audit intervention, employee compensate stickiness of listed companies is significantly reduced, supporting the hypothesis H3. Compared with not audited SOEs, management of audited SOEs reduce employee compensate more in the stage of sales decline. This shows that government audit promotes the dynamic adjustment of labor costs and improves the efficiency of business management.

**Table 7:** The impact of government audit on employee compensate stickiness.

VARIABLES	(1)	(2)	(3)	(4)
	*Lncompen*_*it*_	*Lncompen*_*it*_	*Lncompen*_*it*_	*Lncompen*_*it*_
*Lnsale*_*it*_	0.270***	0.117***	0.141***	0.119***
	(3.842)	(7.963)	(8.682)	(8.411)
*Cap*_*it*_ * *Lnsale*_*it*_	0.003*	0.000		-0.000***
	(1.954)	(0.307)		(-3.102)
*Emp*_*it*_ * *Lnsale*_*it*_	-0.308***		-0.219***	
	(-3.536)		(-2.843)	
*Dec*_*it*−1_ * *Lnsale*_*it*_	-0.099***	-0.106***	-0.100***	-0.105***
	(-5.126)	(-5.514)	(-5.210)	(-5.494)
*Lnsale*_*it*_ * *Dec*_*it*_	-0.440***	-0.146***	-0.170***	-0.148***
	(-2.631)	(-4.111)	(-4.696)	(-4.188)
*List*_*i*_ * *Lnsale*_*it*_ * *Dec*_*it*_	-0.083	-0.082	-0.083	-0.082
	(-0.959)	(-0.943)	(-0.963)	(-0.945)
*Postlist*_*it*−1_ * *Lnsale*_*it*_ * *Dec*_*it*_	0.210**	0.200**	0.202**	0.200**
	(2.031)	(1.961)	(1.979)	(1.962)
*Cap*_*it*_ * *Lnsale*_*it*_ * *Dec*_*it*_	-0.004**	-0.001	-0.000***	
	(-2.127)	(-0.471)	(-3.116)	
*Emp*_*it*_ * *Lnsale*_*it*_ * *Dec*_*it*_	0.309***	0.001**	0.219***	0.001**
	(3.543)	(2.503)	(2.850)	(2.483)
*Dec*_*it*−1_ * *Lnsale*_*it*_ * *Dec*_*it*_	0.182***	0.188***	0.182***	0.186***
	(4.079)	(4.207)	(4.079)	(4.186)
*Year*	control	control	control	control
*Industry*	control	control	control	control
Constant	0.051***	0.039*	0.040**	0.038*
	(2.609)	(1.911)	(1.970)	(1.902)
Observations	9,374	9,374	9,374	9,374
R-squared	0.021	0.019	0.020	0.019

Robust t-statistics in parentheses

*** p<0.01,

** p<0.05,

* p<0.1

### 4.3 Robustness test

#### 4.3.1 PSM sample test

There may be some differences between audited SOEs and not-audited SOEs, which may cause significant differences in excess employees and labor cost stickiness between the treatment group and the control group before government audit, thus reducing the reliability of the difference-in-difference model. Therefore, we retest conclusions using Propensity Score Matching (PSM) sample, and select company size, net profit rate, capital density, inventory proportion, labor intensity, fixed asset size, year, and industry variables for matching. The nearest neighbor matching method is used to find the closest sample for the audited company at a ratio of 1:3 as the control group. The caliper range is set as 0.01, so we get the matching sample based on PSM method. The standardized deviations (% bias) of the matched variables are all less than 10%, and the result of t test does not reject the original hypothesis that there is no systematic difference between the treatment group and the control group. It can be seen that PSM matches the data well. Based on this PSM sample, regression analysis is conducted again, and results are basically unchanged. Fixed effect model is used in the regression process, and dummy variables *Post* are added. In the audited year and subsequent years, the value is 1; otherwise, the value is 0.

#### 4.3.2 The test of sub-sample of listed companies controlled by central enterprises

As listed companies controlled by central enterprises may be subject to more supervision, their excess employees and labor costs may be different from those controlled by other SOEs. In order to further accurately test the robustness of results, we only select listed companies controlled by central enterprises for sample analysis and PSM sample matching. The dummy variables of COD management, dumb variable of SASAC management, year, industry, total operating income and management expense rate are selected for matching. The nearest neighbor matching method is used to find the closest sample for the audited company at a ratio of 1:1 as the control group, so we get the matching sample based on PSM method. Based on this PSM sample, regression analysis is conducted again, and conclusions are almost unchanged.

## 5. Further analysis: Mechanism test

In theory, it is mentioned that the intermediary mechanism of government audit reducing excess employees and labor cost stickiness is that government audit strengthens the incentive mechanism. This section will examine the intermediary mechanism. If the mechanism of government audit reducing excess employees is to strengthen the incentive mechanism, then there will be an inference: for enterprises with higher remuneration incentive, government audit will reduce excess employees more significantly; for enterprises with low remuneration incentive, the reduction effect of government audit on excess employees will be weaken, or even not significant, while the result of equity incentive may be just the opposite. Therefore, we use management incentive indicators to group and test whether strengthening incentive mechanism is an intermediate channel for government audit reducing excess employees and labor cost stickiness. We group the sample from the perspectives of unexpected monetary remuneration, number of shares held by management and Pay-Performance Sensitivity, and use fixed effect model to conduct regression analysis on the sub-samples after grouping.

### 5.1 Monetary compensation not expected by management


$$\begin{array}{l} Pay_{it}=\beta_{0}+\beta_{1}Size_{it}+\beta_{2}Roa_{it}+\beta_{3}Intan_{it}+\beta_{4}Stock_{it}+\sum Year+\sum Industry\\ +\sum Province+\varepsilon_{it} \end{array}$$ (5)

In order to measure the monetary remuneration incentive of management, we use the method of [23] to measure unexpected monetary remuneration using the regression residual *ε*_*it*_ of model (5). In formula (5), *Pay*_*it*_ represents the actual remuneration of management of the *i* company in *t* year, expressed by the natural logarithm of the total remuneration of top three managers. *Intan*_*it*_ is the ratio of intangible assets to total assets at the end of the period. *Roa*_*it*_ is the net profit rate of total assets. *Size*_*it*_ is the natural logarithm of total assets. *Stock*_*it*_ is the logarithm of the number of shares held by management, and *Year* and *Industry* are dummy variables of the year and industry respectively. In addition, due to the difference in consumption levels in different regions, management compensation is different. We add variable *Province* to control the regional effect, and the residual *ε*_*it*_ obtained by regression of the model (5) is the unexpected monetary remuneration, expressed by *Exp_comp*. The higher value represents higher monetary remuneration of management. We add the dummy variable *Dum_comp*. When the regression residual *ε*_*it*_ of the model is positive, meaning the remuneration of management is higher than the normal level, the variable value is 1. When the residual *ε*_*it*_ is negative, the variable value is 0, which means that monetary remuneration fails to compensate management enough and the incentive level is insufficient.

In order to test that strengthening incentive mechanism is an intermediate channel for government audit reducing excess employees and labor cost stickiness, the sample is divided into two groups according to the dummy variable *Dum_comp*. The sample with negative unexpected remuneration is low remuneration incentive group, and the sample with positive unexpected remuneration is high remuneration incentive group. The sub-samples after grouping are regressed and coefficient differences are analyzed to test the impact of government audit on excess employees and labor cost stickiness under different incentive levels. The results are shown in Table 8.

**Table 8:** The test of relationship between government audit and excess employees based on compensate incentive grouping.

VARIABLES	Compensate incentive		Compensate incentive	
	High	Low	High	Low
	*Exemp*1_*it*+1_	*Exemp*1_*it*+1_	*Exemp*2_*it*+1_	*Exemp*2_*it*+1_
*Postlist*_*it*_	-0.005**	-0.004	-0.007**	-0.004
	(-2.041)	(-1.407)	(-2.461)	(-1.527)
*Lnsize*_*it*_	-0.005***	-0.005***	-0.014***	-0.015***
	(-3.629)	(-3.914)	(-10.164)	(-10.573)
*Roa*_*it*_	0.042**	0.083***	0.003	0.046**
	(2.084)	(3.686)	(0.164)	(1.985)
*Threeins*_*it*_	0.003	-0.041***	0.005	-0.038***
	(0.314)	(-4.154)	(0.466)	(-3.828)
*Cap*_*it*_	-0.000	-0.001**	-0.000	-0.001*
	(-0.833)	(-2.061)	(-0.884)	(-1.706)
*Q*_*it*_	-0.002**	-0.001*	-0.003***	-0.002***
	(-2.159)	(-1.649)	(-3.225)	(-2.739)
*Inventorydha*_*it*_	-0.024***	-0.021**	-0.021***	-0.015
	(-3.191)	(-2.289)	(-2.682)	(-1.563)
*Cash*_*it*_	-0.012	-0.002	0.006	0.016**
	(-1.536)	(-0.293)	(0.680)	(2.149)
*Dividend*_*it*_	0.012***	0.005	0.011**	0.003
	(2.869)	(1.312)	(2.483)	(0.887)
*Grow*_*it*_	-0.000	0.001	-0.001	0.000
	(-0.191)	(0.785)	(-0.594)	(0.066)
*Year*	control	control	control	control
*Industry*	.....	.....	.....	.....
Constant	0.110***	0.144***	0.300***	0.338***
	(3.695)	(4.572)	(9.555)	(10.498)
Observations	2,871	3,005	2,871	3,005
R-squared	0.025	0.029	0.069	0.075
Number of code	683	679	683	679

t-statistics in parentheses

*** p<0.01,

** p<0.05,

* p<0.1

It can be seen that the coefficient of *Postlist*_*it*_ for the group with high remuneration incentive is -0.005 and -0.007 respectively at the 5% significance level, and government audit significantly reduces excess employees. For the group with positive *Dum_comp* value and low remuneration incentive, the coefficient of *Postlist*_*it*_ regressed with *Exemp*1_*it*+1_ as the explained variable is negative, but it is not significant. The coefficient of *Postlist*_*it*_ obtained by regression with *Exemp*2_*it*+1_ as the explained variable is also negative, but not significant. It can be seen that government audit doesn’t significantly reduce excess employees. The regression result obtained by PSM samples grouping are basically consistent and are not listed here. The above results are completely consistent with the inference that government audit reduces excess employees by strengthening incentive mechanism of enterprises, which confirms the intermediary effect of strengthening incentive mechanism.

### 5.2 Number of shares held by management

We measure the incentive level based on the number of shares held by management. The sample is divided into management shareholding group and no management shareholding group for grouping regression. The results are shown in Table 9. It can be seen that when *Exemp*1_*it*+1_ is taken as the explanatory variable, the coefficient of *Postlist*_*it*_ is negative at the significance level of 1% for the group without management shareholding, while the coefficient of *Postlist*_*it*_ is not significant in the group with management shareholding. When *Exemp*2_*it*+1_ is taken as the explanatory variable, the coefficient of *Postlist*_*it*_ is negative at the significance level of 1% for the group without management shareholding, while the coefficient of *Postlist*_*it*_ is not significant for the group with management shareholding. These indicate that government audit significantly reduces excess employees for companies without management equity incentive. This is completely consistent with the inference that government audit can reduce excess employees by strengthening incentive mechanism, and confirms the intermediary mechanism of strengthening incentive mechanism.

**Table 9:** The test of relationship between government audit and excess employees based on management shareholding grouping.

VARIABLES	Management shareholding		Management shareholding	
	Low	High	Low	High
	*Exemp*1_*it*+1_	*Exemp*1_*it*+1_	*Exemp*2_*it*+1_	*Exemp*2_*it*+1_
*Postlist*_*it*_	-0.005***	-0.001	-0.006***	-0.002
	(-2.727)	(-0.148)	(-3.115)	(-0.255)
*Lnsize*_*it*_	-0.002**	-0.021***	-0.012***	-0.031***
	(-2.519)	(-3.917)	(-12.882)	(-5.542)
*Roa*_*it*_	0.083***	0.047	0.044***	0.025
	(5.799)	(0.609)	(2.944)	(0.316)
*Threeins*_*it*_	-0.005	-0.085***	-0.004	-0.102***
	(-0.763)	(-2.734)	(-0.536)	(-3.138)
*Cap*_*it*_	-0.001**	-0.005**	-0.001**	-0.007**
	(-2.356)	(-2.124)	(-2.098)	(-2.580)
*Q*_*it*_	-0.001	-0.003*	-0.001***	-0.004*
	(-1.500)	(-1.654)	(-2.693)	(-1.899)
*Inventorydha*_*it*_	-0.023***	0.006	-0.021***	0.026
	(-4.153)	(0.178)	(-3.542)	(0.777)
*Cash*_*it*_	-0.008	-0.001	0.010	0.029
	(-1.401)	(-0.048)	(1.624)	(1.197)
*Dividend*_*it*_	0.007***	0.029*	0.006**	0.029*
	(3.100)	(1.706)	(2.464)	(1.656)
*Grow*_*it*_	0.001*	-0.010*	0.001	-0.012**
	(1.746)	(-1.943)	(0.963)	(-2.080)
*Year*	control	control	control	control
*Industry*	.....	.....	.....	.....
Constant	0.053***	0.538***	0.257***	0.752***
	(2.578)	(4.511)	(11.938)	(6.080)
Observations	5,485	542	5,485	542
R-squared	0.024	0.141	0.064	0.230
Number of code	925	223	925	223

Robust t-statistics in parentheses

*** p<0.01,

** p<0.05,

* p<0.1

### 5.3 Pay-performance sensitivity

Drawing on the existing literature [24], we measure the degree of incentive by Pay-Performance Sensitivity. Generally speaking, higher Pay-Performance Sensitivity represents higher incentive degree of compensation system to senior executives. We use Pay-Performance Sensitivity grouping to test the intermediary effect of government audit strengthening the incentive mechanism. As it is difficult to accurately quantify Pay-Performance Sensitivity, we refer the measurement method of [25], and use dummy variables to describe Pay-Performance Sensitivity, which is divided into two categories according to whether the performance and compensate are greater or lower than the median, thus forming 2×2 cases. If both the executive compensation and performance in *t* period are higher than the median, or both the executive compensation and performance are lower than the median, it is considered that executive compensation is sensitive to performance, and the dummy variable *Cer* of Pay-Performance Sensitivity is 1, and *Cer* of the other two cases is 0. One study [25] believes that the variable is applicable to Chinese enterprises as a measurement of Pay-Performance Sensitivity. We use *Cer* to group the sample for regression, and the results are shown in Table 10.

**Table 10:** The test of relationship between government audit and excess employees based on Pay-Performance Sensitivity grouping.

Variables	*Exemp*1_*it*+1_		*Exemp*2_*it*+1_	
	*Cer* = 0	*Cer* = 1	*Cer* = 0	*Cer* = 1
*Postlist*_*it*_	-0.002	-0.005**	-0.002	-0.006**
	(-0.492)	(-2.019)	(-0.601)	(-2.383)
*Lnsize*_*it*_	-0.006***	-0.002	-0.016***	-0.012***
	(-3.562)	(-1.278)	(-9.347)	(-8.520)
*Roa*_*it*_	0.077**	0.036*	0.060*	0.008
	(2.548)	(1.714)	(1.945)	(0.376)
*Threeins*_*it*_	-0.003	-0.042***	-0.005	-0.041***
	(-0.244)	(-4.981)	(-0.377)	(-4.535)
*Cap*_*it*_	-0.001	-0.004***	-0.001	-0.005***
	(-0.727)	(-5.093)	(-0.776)	(-5.333)
*Q*_*it*_	-0.001	-0.001	-0.002*	-0.001**
	(-0.991)	(-1.121)	(-1.745)	(-2.041)
*Inventorycha*_*it*_	-0.016*	-0.016*	-0.012	-0.012
	(-1.652)	(-1.758)	(-1.208)	(-1.219)
*Cash*_*it*_	-0.026***	0.013*	-0.007	0.032***
	(-2.743)	(1.921)	(-0.706)	(4.371)
*Dividend*_*it*_	0.017*	0.011**	0.019**	0.012***
	(1.921)	(2.577)	(2.035)	(2.664)
*Grow*_*it*_	0.001	0.002	0.001	0.001
	(0.742)	(1.577)	(0.415)	(0.885)
*Year*	control	control	control	control
*Industry*	.....	.....	.....	.....
Constant	0.146***	0.068**	0.361***	0.278***
	(3.742)	(2.245)	(9.053)	(8.766)
Observations	2,363	2,994	2,363	2,994
R-squared	0.031	0.043	0.088	0.094
Number of code	671	752	671	752

Robust t-statistics in parentheses

*** p<0.01,

** p<0.05,

* p<0.1

From Table 10, we find that the coefficient of *Postlist*_*it*_ is not significant for the group with low Pay-Performance Sensitivity, while the coefficient of *Postlist*_*it*_ is significant for the group with high Pay-Performance Sensitivity. The regression results by PSM samples grouping are basically consistent and are not listed here. The above results are in line with the inference that government audit reduces excess employees by strengthening incentive mechanism, which confirms the intermediary effect of strengthening incentive mechanism.

## 6. Conclusion and enlightenment

This paper uses the quasi natural experiment provided by the audit of SOEs implemented by the National Audit Office to examines the impact of government audit on employee efficiency and labor cost stickiness. The empirical results show that in the year after the intervention of government audit, excess employees and labor cost stickiness of SOEs significantly decrease, which confirms the supervision and governance function of government audit on employee efficiency of SOEs. Moreover, after the implementation of government audit, labor costs of audited SOEs don’t decrease significantly, which shows from the side that the compensation system of SOEs conforms to market mechanisms, and there is no problem of excessive employee compensation in China. Further research finds that the impact of government audit on employee efficiency mainly exists in the samples with more unexpected monetary remuneration, less number of shares held by management and higher pay-performance sensitivity, which confirms the intermediary role of government audit on strengthening incentive mechanism. All the above conclusions pass the robustness test. The research in this paper reveals the changes in excess employees and labor cost stickiness of SOEs after the intervention of government audit, which helps us better understand the implementation effect of government audit, enrich the relevant literature on the impact of government audit on SOEs, and provide empirical support for the full coverage of government audit.

Government audit strengthens management incentive mechanism, which enables management to have higher motivation to create profits and actively carry out cost management. However, senior executives of SOEs don’t blindly cut labor costs, but flexibly adjust wages and benefits according to the economic situation and operating income, which reduces labor cost stickiness and business risk of enterprises. Management make great efforts to improve enterprise performance, which reduces labor cost stickiness, but not reduced labor costs, indicating that the remuneration of SOEs is relatively reasonable. In a word, after government audit, labor cost stickiness decreases, the number of excess employees decreases, and the employees efficiency increases. However, we should also realize that after the intervention of government audit, employees are retained during bad economic period, and enterprises overcome the difficulties by reducing their salaries, which is different from that of developed countries. We suggest that SOEs should establish partial elimination system, recruit new employees, and cut jobs during bad economic times. In the case of layoffs, enterprises should stabilize core employees through remuneration incentives, reduce the fluctuation of remuneration, and retain outstanding talents.

## Supporting information

S1 Appendix (DOCX)

S1 File (DTA)
