# Supplementary material for: Government audit, employee efficiency and labor cost stickiness
Source: PLoS One. 2023 Sep 1;18(9):e0291014. doi: 10.1371/journal.pone.0291014 (PMC10473528; doi:10.1371/journal.pone.0291014)
Supplement: S1 Appendix — (DOCX) [file pone.0291014.s001.docx]

**Appendix** Table 1 Description of Variables in this Document

| Variable Symbol | Variable name | Variable definition |
| --- | --- | --- |
| **$Num$** | Number of employees | Number of employees of listed companies |
| **$Emp$** | Staff size | Number of employees divided by total assets at the end of the year and multiplied by 100000 |
| **$Exemp1$** | Excess employees | Residual calculated according to formula (0) (regardless of year and industry) |
| **$Exemp2$** | Excess employees | Residual calculated according to formula (0) (by industry, regardless of year) |
| $List$ | Dummy variable of government audit | When the listed company is audited by the National Audit Office, 1 is taken; otherwise, 0 is taken. |
| $Postlist$ | Time variable of government audit | The listed company is audited in the current year and subsequent years, 1 is taken; otherwise, 0 is taken. |
| **$Lev$** | financial leverage | Total liabilities divided by total assets at the end of the period |
| **$Lnsize$** | Enterprise scale | Natural logarithm of total assets at the end of the period |
| **$Roa$** | Net profit rate of assets | Current net profit divided by total assets at the end of the period |
| **$Grow$** | Sales revenue growth rate | The current sales minus the previous sales, and then divided by the previous sales |
| **$Ppe$** | Scale of fixed assets | Fixed assets divided by total assets at the end of the period |
| **$Age$** | Time of listing | The number of days from the company's listing date to the end of the current period divided by 360 |
| **$Cap$** | Capital intensity | It is expressed by the ratio between the total assets and operating income of the enterprise |
| **$Q$** | Tobin Q value | The sum of the market value of stocks and the market value of debts at the end of the year, divided by the total assets of the enterprise |
| **$Cash$** | Cash ratio | Cash and its equivalents at the end of the period divided by average total assets |
| **$Dividend$** | Dividends per share | Cash dividends per share |
| $Threeins$ | Shareholding ratio of institutional investors | Ratio of total shares held by top three institutional investors to outstanding shares |
| **$Invchange$** | Changes in inventories | The inventory at the end of the period minus the inventory at the beginning of the period, and then divided by the average total assets |
|  |  |  |
| **$Labcost$** | Labor cost | Total compensation paid to employees minus total executive compensation |
| **$Labcost1$** | Labor cost | Total compensation paid to employees minus total executive compensation，and divide by the total number of employees excluding the number of executives to get the average compensation of employees, and then take the natural logarithm. |
| **$Labcost2$** | Labor cost | The natural logarithm of "cash paid to and for employees" disclosed in the cash flow statement. |
| **$Labcost3$** | Labor cost | Divide the "cash paid to and for employees" disclosed in the cash flow statement by the total operating costs |
| **$Labcost4$** | Labor cost | Divide the "cash paid to and for employees" disclosed in the cash flow statement by the average total assets |
|  |  |  |
| **$Lnlabcost$** | Labor cost growth rate | The amount of "cash paid to and for employees" disclosed in the cash flow statement is divided by the amount of the previous period, and then the natural logarithm is taken |
| **$Lncompen$** | Employee compensation change rate | Total compensation paid to employees minus total executive compensation, and divide by the total number of employees excluding the number of executives to get the average compensation of employees. Divide the current period by the previous period, and then take the natural logarithm. |
| $Emp_{it}$ | Labor intensity | Divide the current amount of "cash paid to and for employees" disclosed in the cash flow statement by the operating income |
| $Dec_{it}$ | Dummy variable of business income decline | If the sales amount in the current period is lower than that in the previous period, it is 1; otherwise, it is 0. |
| $Year$ | Annual dummy variable | Controlling annual effect |
| $Industry$ | Industry dummy variable | Controlling industry effect |
